# Discrepancy between clinical and pathological staging of laryngeal carcinoma: a dilemma to be solved

**DOI:** 10.1007/s00405-024-08506-2

**Published:** 2024-02-12

**Authors:** Ali Tawfik, Ahmed Musaad Abd El-Fattah, Amany Hassan, Fatma Ahmad Helal, Hisham Atef Ebada

**Affiliations:** 1 Department of Otorhinolaryngology, Mansoura University, Mansoura, 35511 Egypt; 2 Pathology, Mansoura University, Mansoura, Egypt

**Keywords:** TNM, Staging, Clinical, Pathological, Laryngeal carcinoma, Survival

## Abstract

**Objectives:**

The aim of this study was to investigate the degree of discrepancy between the clinical and pathological staging of laryngeal carcinoma, and the potential impact of this discrepancy on the outcomes and prognosis.

**Methods:**

This study was conducted on 127 patients who underwent total laryngectomy over five years (October 2016–October 2021). Data collected from pretherapeutic clinical staging regarding the extent of the tumor affection of different laryngeal subsites was compared to the postsurgical pathological assessment.

**Results:**

Overall, 12 out of 127 patients (9.4%) in the current study, were clinically over-staged from T3 to T4 due to radiological diagnosis of tumor infiltration of laryngeal cartilages that proved pathologically to be free of tumor. Additionally, discordance in the N stage was found in 12.6% (n = 16). However, stage discrepancy did not have a significant impact on the prognosis and survival.

**Conclusion:**

Discordance between clinical and pathological TNM staging of laryngeal carcinoma may affect the decision making and the choice of the treatment options. Some improvement can be probably achieved with advancements and higher accuracy of the preoperative diagnostic tools.

## Introduction

The TNM classification is considered to be the most reliable system defining the extent of the primary tumor and its regional and distant metastases. Correct TNM staging is fundamental for therapeutic decisions and patient counselling. It also plays an important role as a prognostic factor [1].

Clinical TNM (cTNM) classification is based on the findings of physical examination, endoscopy, and imaging. To determine pathological TNM (pTNM) classification, a detailed histopathological analysis of surgically removed tissue is necessary [1]. The appropriate management of laryngeal carcinoma depends mainly on accurate pre-operative clinical TNM staging [2].

Laryngeal cartilages invasion greatly influences the decision making whether to perform surgery or to adopt the organ preservation strategy (radio-chemotherapy) [3, 4]. Tumors extending through the thyroid cartilage into the superficial soft tissues of the neck are staged as T4a and may require total laryngectomy [5]. On the other hand, potentially organ-preserving treatment may still be performed in case of focal cartilage thyroid invasion without full-thickness extension (T3) [6].

Recent studies showed that none of the modern imaging modalities can accurately verify the extent of the primary tumor accurately or to prove the presence of metastases in regional lymph nodes [7]. This is the main reason why in some patients, a discordance between cTNM and pTNM classification can be demonstrated [1]. The aim of this work is to investigate the degree of discrepancy between the clinical and pathological staging of laryngeal carcinoma, and the potential impact of this discrepancy on the outcomes and prognosis.

## Patients and methods

This is a prospective study that was conducted in the Otorhinolaryngology Department, Mansoura University, Egypt over five years (October 2016–October 2021). The study included 127 patients with advanced (T3 and T4) laryngeal carcinoma who underwent total laryngectomy. Patients who received preoperative radio/chemotherapy were excluded from the study. Informed written consents were obtained from all participants, and the study was approved by the Mansoura Faculty of Medicine Institutional Research Board (MFM-IRB: MS.19.09.792).

### Clinical staging

In the outpatient setting, the larynx was assessed using the flexible nasal laryngoscope and rigid telescopes. Specific findings were noted in patients’ records including the site of the primary tumor, involved subsites, extension to adjacent structures, vocal fold mobility (normal, impaired, or fixed), and patency of the airway. Neck palpation was performed to assess the condition of the lymph nodes.

Imaging was performed before operative endoscopy and biopsy to obtain images before potential edema and distortion from biopsy and manipulation of the larynx. Neck ultrasonography, contrast-enhanced CT (CECT) as well as magnetic resonance imaging (MRI) were performed for all patients (n = 127).

**Fig. 1:**
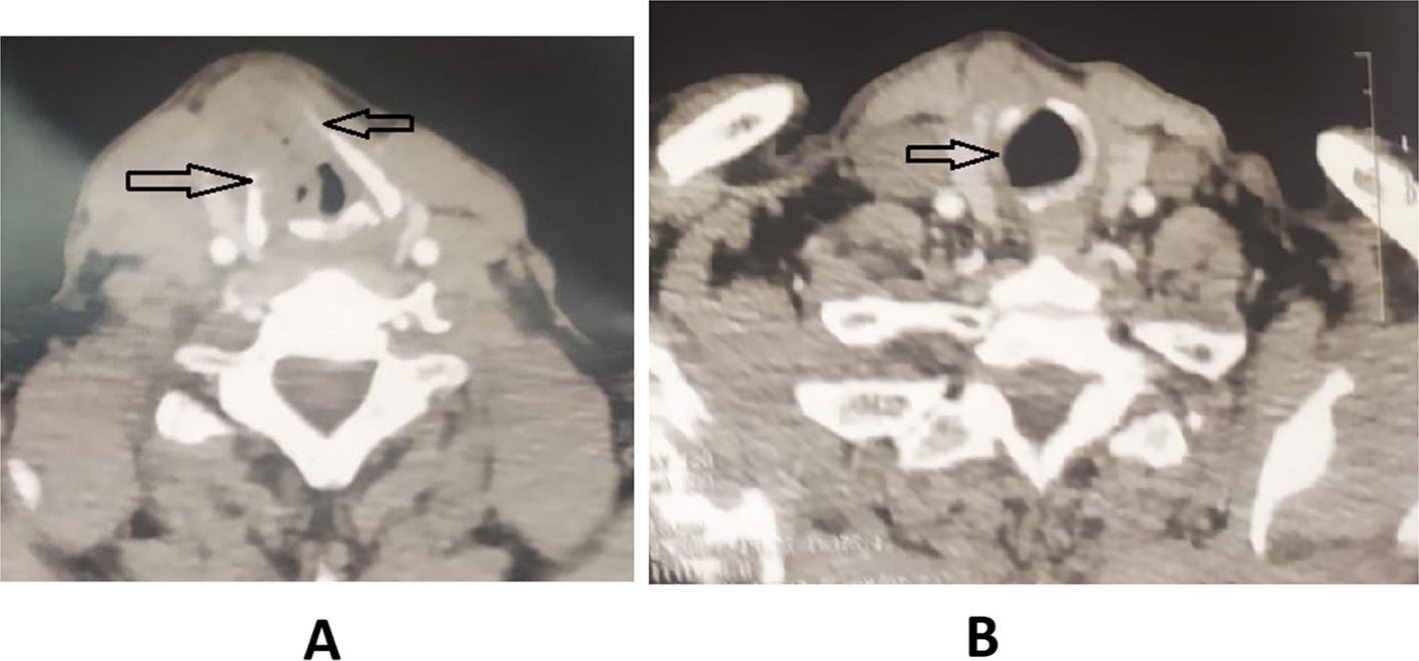
CT scan showing laryngeal cartilages invasion. **A** Thyroid cartilage invasion (arrows). **B** Cricoid cartilage invasion (arrow)

According to American National Comprehensive Cancer Network (NCCN) clinical practice guidelines in oncology for head and neck cancers (version 3.2019–September 16, 2019) [8], erosion of the inner cortex and full-thickness infiltration of thyroid cartilage were staged as cT3 and cT4, respectively. The erosion of the inner cortex was defined as any focal cartilage defect in close proximity to the neoplastic mass. Full-thickness thyroid cartilage invasion was defined as tumor-like tissue replacing the thyroid cartilage or extending in extra-laryngeal soft tissues on post-contrast images. Invasion of the arytenoid and cricoid was diagnosed when sclerosis or cartilage erosion were detected in an area contacting the tumor tissue (Fig. 1).

All patients underwent an endoscopic examination under general anesthesia. Direct laryngoscopy allowed to examine the larynx in greater detail, palpate the larynx, and obtain a biopsy for histologic analysis.

### Pathological (postoperative) staging

Total laryngectomy and neck dissection was performed for all patients (whether therapeutic neck dissection in N + patients, or elective neck dissection for N0 patients). Laryngeal and neck dissection specimens, that were removed by were examined to determine the extension of the tumor and the affection of the different laryngeal subsites.

**Fig. 2:**
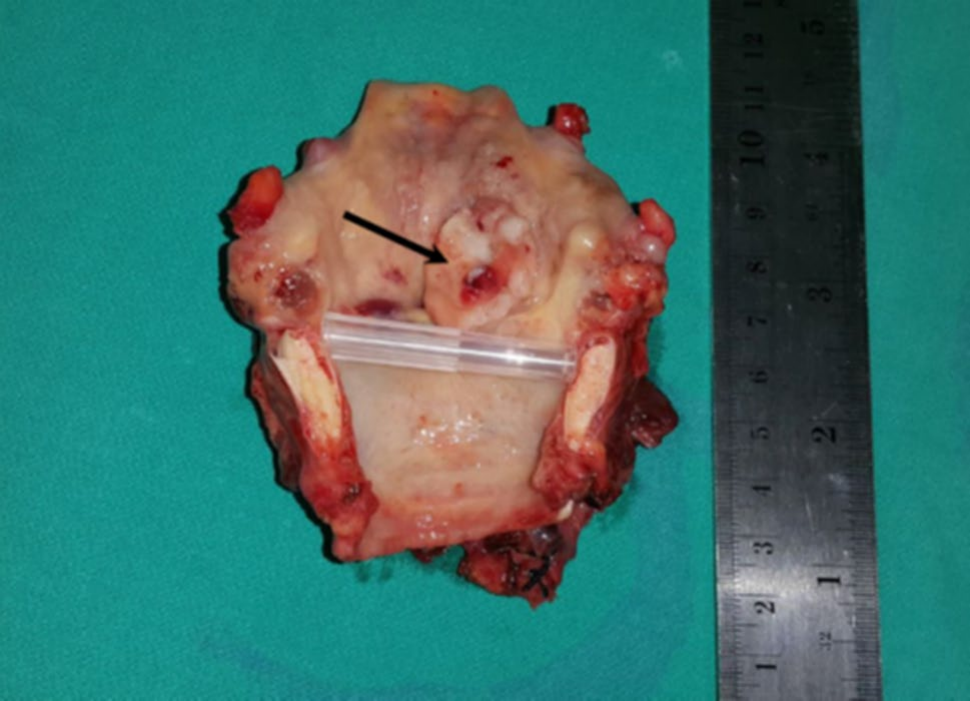
Postoperative photo of a larynx with right transglottic carcinoma (arrow)

The resected larynx was opened in the posterior midline and the gross appearance with the extension of the tumor was described and photographed (Fig. 2). The entire laryngeal specimen was subjected to fixation in 10% formalin–saline for a minimum of 3 days and decalcified in 30% formic acid/citric acid mixture for 7–10 days depending on the degree of cartilage calcification. Multiple vertical, coronal, or sagittal serial sections, approximately 3 mm thick, were cut with the microtome at right angles to the mucosa. The sections were stained with hematoxylin/eosin and subjected to detailed microscopic scrutiny. The pathologist had no prior knowledge of the initial clinical diagnosis and the pretherapeutic staging and tumor extension.

### Data collection

All subsites of the larynx were assessed regarding tumor infiltration. These subsites included the epiglottis, aryepiglottic fold, medial pyriform sinus, false vocal folds, ventricle, true vocal folds, anterior commissure, arytenoids, subglottis, pre-epiglottic space, paraglottic space, thyroid cartilage, and cricoid cartilage.

Data collected from each step of pretherapeutic clinical staging (laryngoscope in outpatient, direct laryngoscope under general anesthesia and imaging) regarding the extent of the tumor and affection of different laryngeal subsites was recorded, documented, and tabulated. The accuracy of the pretherapeutic clinical staging was evaluated by correlation between it and postsurgical pathological staging. Similarly, the clinical staging of cervical lymph nodes was compared to the postoperative pathological staging.

Additionally, survival outcome was assessed by considering patients as being alive with and without oncologic disease; dead with local, regional or distant disease; dead without oncologic disease; and finally, lost to FU. The cutoff point for statistical analysis was October 2023, encompassing a minimum FU of 24 months. Overall survival and disease specific survival curves were calculated using the Kaplan–Meier method, and statistical significance was determined by the Log-Rank test.

## Results

One hundred twenty-seven patients were included in this study. The age of the study population ranged from 45 to 76 years (mean: 58.87). One hundred twenty-five patients were males (98.4%), and 2 patients were females (1.6%).

The clinical (pre-operative) T staging of the study population was T3 in 47.2% (n = 60) and T4 in 52.8% (n = 67). The preoperative N staging was N0 in 56 patients (44.1%), N1 in 41 patients (32.3%), N2 in 27 patients (21.3%), and N3 in 3 patients (2.3%).

Table 1 shows the correlation between clinical and pathological staging. It was noticed that the number of patients who showed invasion of arytenoids, aryepiglottic folds, and pyriform fossa in CT scan were much higher than detected by endoscopy in these subsites. Arytenoids were affected in 91 patients (71.7%) by imaging, while in only 51 patients (40.2%) by direct endoscopy. Similarly, aryepiglottic folds were affected in 67 patients (52.8%) by imaging, and in 42 patients (33.1%) by direct laryngoscopy. Lastly, the pyriform sinus was invaded in 42 patients (33.1%) by imaging, but in 25 (19.7%) with direct laryngoscope.

**Table 1:** Correlation between outpatient laryngoscopy, direct laryngoscopy and imaging findings with pathology

Variables	Outpatient laryngoscopy	Direct laryngoscopy	Imaging findings	Pathology	p value
**Epiglottis**					P1 = 1
Positive Negative	34 (26.8%) 93 (73.2%)	34 (26.8%) 93 (73.2%)	34 (26.8%) 93 (73.2%)	34 (26.8%) 93 (73.2%)	P2 = 1 P3 = 1
**Aryepiglottic fold**					P1 = 0.273
Positive Negative	34 (26.8%) 93 (73.2%)	42 (33.1%) 85 (66.9%)	67 (52.8%) 60 (47.2%)	50 (39.4%) 77 (60.6%)	P2 = 0.592 P3 = 0.301
**Pyriform sinus**					P1 = 0.197
Positive Negative	34 (26.8%) 93 (73.2%)	25 (19.7%) 102 (80.3%)	42 (33.1%) 85 (66.9%)	17 (13.4%) 110 (86.6%)	P2 = 0.488 P3 = 0.067
**False vocal cords**					P1 = 0.292
Positive Negative	67 (52.8%) 60 (47.2%)	69 (54.3%) 58 (45.7%)	76 (59.8%) 51 (40.2%)	84 (66.1%) 43 (33.6%)	P2 = 0.292 P3 = 0.592
**Ventricles**					–
Positive Negative	–	92 (72.4%) 35 (27.6%)	92 (72.4%) 35 (27.6%)	102 (80.3%) 25 (19.7%)	P2 = 0.542 P3 = 0.542
**True vocal folds**					P1 = 0.038*
Positive Negative	89 (70.1%) 38 (29.9%)	89 (70.1%) 38 (29.9%)	119 (93.7%) 8 (6.3%)	119 (93.7%) 8 (6.3%)	P2 = 0.038* P3 = 1.0
**Anterior commissure**					P1 = 0.067
Positive Negative	85 (66.9%) 42 (33.1%)	85 (66.9%) 42 (33.1%)	85 (66.9%) 42 (33.1%)	110 (86.6%) 17 (13.4%)	P2 = 0.067 P3 = 0.067
**Arytenoids**					P1 = 0.592
Positive Negative	51 (40.2%) 76 (59.8%)	51 (40.2%) 76 (59.8%)	91 (71.7%) 36 (28.3%)	42 (33.1%) 85 (66.9%)	P2 = 0.592 P3 = 0.002*
**Subglottis**					–
Positive Negative	–	93 (73.2%) 34 (26.8%)	91 (71.7%) 36 (28.3%)	101 (79.6%) 26 (20.4%)	P2 = 0.542 P3 = 0.542
**Pre-epiglottic space**					–
Positive Negative	–	–	44 (34.6%) 83 (65.4%)	43 (33.9%) 84 (66.1%)	– P3 = 1.0
**Paraglottic space**					–
Positive Negative	–	–	92 (72.4%) 35 (27.6%)	124 (97.6%) 3 (2.4%)	– P3 = 0.002*
**Thyroid cartilage**					–
Positive Negative	–	–	90 (70.9%) 37 (29.1%)	87 (68.6%) 40 (31.4%)	– P3 = 0.573
**Cricoid cartilage**					–
Positive Negative	–	–	56 (44.1%) 71 (55.9%)	36 (28.3%) 91 (71.7%)	– P3 = 0.108
**Lymph nodes**					–
Positive (N1, N2, N3) Negative (N0)	–	–	71 (55.9%) 56 (44.1%)	63 (49.6%) 64 (50.4%)	– P3 = 0.486

Chi square test was used

*Significant p ≤ 0.05, P1: compare outpatient laryngoscope with pathology, P2: compare direct laryngoscope with pathology, P3: compare radiology with pathology

After laryngectomy, the specimens were pathologically examined, and the laryngeal subsites were individually assessed for tumor invasion. The neck dissection specimens were also pathologically examined. The accuracy of the clinical staging was assessed using the post operative (pathological) staging as the reference.

The number of patients who showed invasion of the epiglottis were the same when assessed by office endoscopy, operative endoscopy, imaging, and pathology. Similarly, the pre-epiglottic space was diagnosed to be invaded by imaging in 44 patients (34.6%) and the pathological examination confirmed the invasion in 43 patients (p = 1).

Other subsites showed a statistically significant discordance between clinical and pathological staging. Invasion of the true vocal folds was detected in 89 patients (70.1%) by both office endoscopy and operative endoscopy, and it was proved in 119 patients (93.7%) pathologically (p = 0.038). Similarly, arytenoid cartilage invasion was detected in CT in 91 patients (71.7%) and in only 42 patients (33.1%) pathologically. Consequently, CT scan over-estimated arytenoid cartilage invasion significantly. Invasion of para-glottic space was diagnosed by imaging in 92 patients (72.4%) but it was proved pathologically in 124 (97.6%) of the specimens (p = 0.002).

Invasion of the pyriform sinus was detected clinically in 34 patients (26.8%) by office endoscopy, in 25 (19.7%) by operative endoscopy, and in 42 patients (33.1%) by imaging. Pathology, on the other hand, proved affection in only 17 specimens (13.4%) indicating that clinical evaluation over-estimated pyriform affection. However, it was statistically not significant (p = 0.067%). Similarly, imaging studies over-diagnosed cricoid cartilage invasion in 44.1% (n = 56), when compared to pathological diagnosis (28.3%—n = 36) but it was statistically insignificant (p = 0.108).

Regarding N staging, 12 out of 71 patients (16.9%) who were pre-operatively diagnosed as clinically positive necks, showed negative pathology. On the other hand, 4 out of 56 patients (7.1%) who underwent elective neck dissection for clinically negative necks showed positive pathology in the lymph nodes. Consequently, the overall discrepancy in the N staging was 12.6% (16 patients out of 127).

Overall, 12 out of 127 patients (9.4%) in the current study, were clinically over-staged from T3 to T4. This over-staging occurred due to radiological diagnosis of tumor infiltration of laryngeal cartilages that proved pathologically to be free of tumor. Inaccuracy in clinical assessment of tumor invasion of other laryngeal subsites in the form of over-estimation or under-estimation, has been reported in this study, however this did not affect the overall staging.

The sensitivity, specificity, positive predictive value, negative predictive value, and accuracy of imaging in detecting cartilage invasion was calculated as shown in Table 2.

**Table 2:** Diagnostic accuracy of radiology as compared to pathology

	Sensitivity	Specificity	PPV	NPV	Accuracy
Thyroid cartilage	80%	40%	72.7%	50%	66.7%
Cricoid cartilage	75%	63.6%	42.8%	87.5%	66.7%
Arytenoids	100%	40%	45.5%	100%	60%

**Fig. 3:**
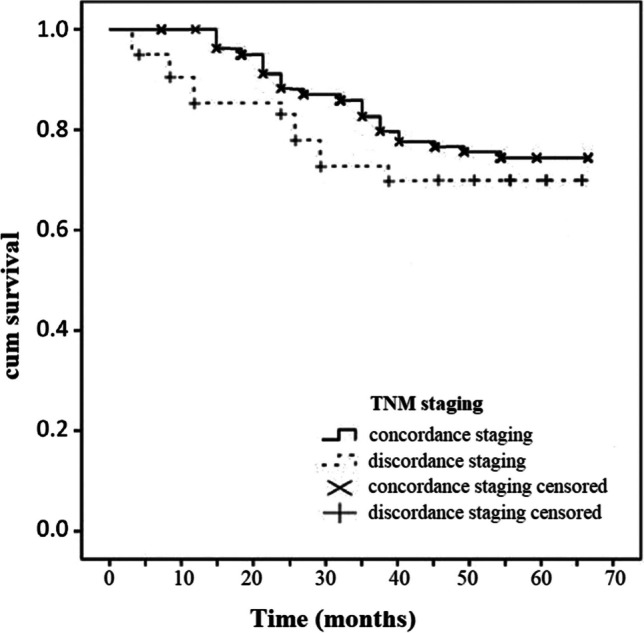
Overall Survival Kaplan–Meier curves for TNM stage concordance and discordance. Long-rank test *p* = 0.235

**Fig. 4:**
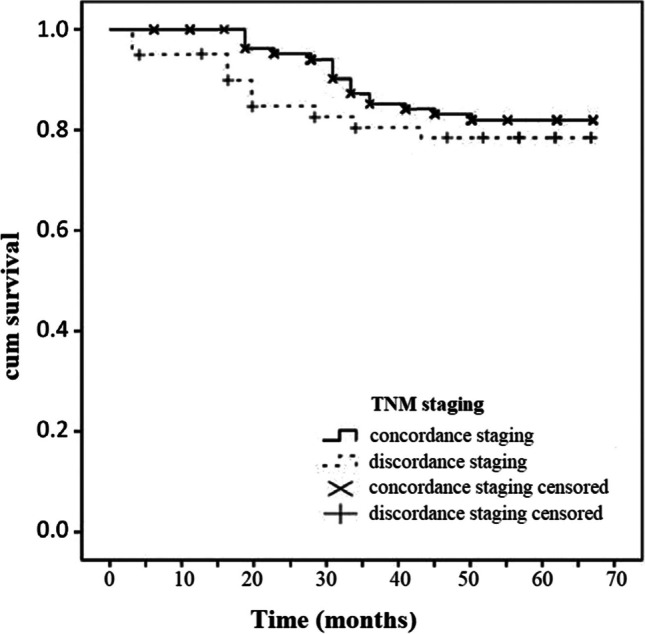
Disease specific Kaplan–Meier curves for TNM stage concordance and discordance. Long-rank test *p* = 0.356

Based on the multidisciplinary team decisions, all patients in the current series received postoperative adjuvant radiotherapy. Regarding survival outcomes, 5-year overall survival was 67.2%, and 5-year disease specific survival was 78.9%. The staging discrepancy did not have a significant impact on the survival rates. Figures 3 and 4 show the overall survival and disease specific survival curves, respectively. *P* values were 0.235 and 0.356, respectively, with no statistically significant differences between stage concordance and stage discordance patients.

## Discussion

Accurate preoperative TNM staging of the tumor is necessary for the proper treatment of laryngeal cancer [2]. Clinical staging relies on endoscopic and imaging findings for assessment of different laryngeal subsites. Endoscopic examination provides an accurate assessment of the surface extent of the tumor but not its depth of penetration or its relationship to deep structures. Cross sectional imaging allows the evaluation of the deep extent of the tumor [2, 9].

The accuracy of preoperative clinical examination, endoscopy and imaging in glottic cancer was studied by Zbären et al. [10, 11]. Their studies demonstrated inability to detect cartilage invasion and extra-laryngeal tumor extension by the clinical evaluation, and consequently a low staging accuracy (55%). The combination of clinical evaluation and either computed tomography (CT) or magnetic resonance imaging (MRI) resulted in a significantly improved staging accuracy (80 vs 87%, respectively).

Pietragalla et al. [12] performed a study on 113 patients with laryngeal carcinoma to evaluate the accuracy of CT scan in detecting cartilage invasion. They reported that perfect correlation between imaging and histopathology when there was full thickness thyroid cartilage invasion with extra-laryngeal tumor extension. On the other hand, when there was no evidence of extra-laryngeal spread, there was poor correlation between CT and histopathological evaluation (sensitivity 77.8%, specificity 87.1%, positive predictive value (PPV) 63.6%, negative predictive value (NPV) 93.1%, accuracy 85%). In the current study the accuracy of CT scan for detection of thyroid cartilage invasion was 66.7% (sensitivity 80%, specificity 40%, PPV 72.7%, NPV 50%).

Regarding arytenoid cartilage, sclerosis of this cartilage in CT scan showed poor correlation with histopathological evaluation. The low specificity of the CT is due to inability to differentiate between normal ossification and sclerosis which occurs due to neoplastic infiltration. Additionally, cartilage sclerosis may be included by contact with adjacent tumor tissue without true cartilage invasion [13, 14]. Furthermore, arytenoid cartilages may show various degrees of sclerosis and ossification in normal conditions, with frequently asymmetric and inhomogeneous patterns on CT [12]. In the series of Pietragalla et al. [12], the accuracy of CT in detection or arytenoid invasion was 67.5% (sensitivity 71.4%, specificity 66.7%, PPV 31.2%, NPV 91.7%). In the current series the accuracy was 60% (sensitivity 100%, specificity 40%, PPV 45.5%, NPV 100%).

Agada et al. [2] performed a study to evaluate the sensitivity of CT scan in detecting cartilage invasion in laryngeal squamous cell carcinoma. They reported that 14 of 38 were accounted for by the use of arytenoid cartilage sclerosis with adjacent tumor as the only radiological criteria for cartilage invasion. This had erroneously upstaged 14 tumors to a T4 stage. This radiological sign of neoplastic cartilage invasion had a sensitivity of 62%, a specificity of 42% and a positive and negative predictive value of 31 and 73%, respectively, in this series.

In this work, 9.4% of patients (n = 12), were clinically over-staged from T3 to T4. Over-diagnosis of cartilage invasion by CT and MRI was the cause of this over-staging in these patients. Li and Chen [15] concluded that the low positive predictive value of CT in detecting full-thickness invasion of thyroid cartilage can result in T4 tumor over-staging. Similarly, Pietragalla et al. [12], reported that CT resulted in downstaging in 5% of patients (cT3 vs. pT4a) and upstaging in 10% of patients (cT4a vs. pT3), wherein the tumor infiltration was limited to the inner cortex in histological examination.

A frequent failure of pre-therapeutic staging of laryngeal cancer involving anterior commissure was confirmed by Foucher et al. [16], who concluded that 25% of cT2 and 33% of cT3 laryngeal tumors were reclassified to pT4 after the histopathological examination. In the current series, pre-therapeutic assessment revealed anterior commissure invasion in 66.9% (n = 85). On the other hand, post-operative pathological assessment revealed invasion in 86.6% (n = 110), with no statistically significant difference.

Reviewing the current literature showed only a limited number of studies focusing on the accuracy of cTNM and pTNM staging in head and neck cancer patients. Koch et al. [17] compared cTNM and pTNM classification in a large group of 501 patients with head and neck cancer. A disparity between cTNM and pTNM staging was proven in almost 50% of cases. According to the author, both cTNM and pTNM classification showed a strong association between the stage and overall survival.

Celakovsky et al. [1] conducted a study on 124 patients with laryngeal carcinoma over 10 years, to evaluate the discordance between clinical and pathological staging. They found disparity in at least one component of TNM staging in 40 patients (32%). Moreover, this discordance had significant negative influence on the locoregional control, cancer relapse and the disease-specific survival rate. Celakovsky et al. [1] found a disparity between cTNM and pTNM classification in 32% of laryngeal cancer patients. In the current study, discordance in the T stage was found in 9.4% of patients (12/127), and in the N stage in 12.6% (n = 16). However, this disparity in our study did not have a statistically significant impact on the overall outcome and survival rates.

Limitations of the current study include the relatively small number of patients and short follow-up period. Therefore, further multicenter studies on a larger number of patients and long follow up periods are warranted. Additionally further studies on the more recent imaging modalities such as PET/MRI or diffusion weighted MRI [18, 19], are needed to verify the accuracy of these modalities, and to decrease the discrepancy between clinical and pathological staging of laryngeal carcinoma.

## Conclusion

Discordance between clinical and pathological TNM staging of laryngeal carcinoma may affect the decision making and the choice of the treatment options. Additionally, it may affect the prognosis and survival outcomes. Some improvement can be probably achieved with advancements and higher accuracy of the preoperative diagnostic tools.

## Data Availability

The data supporting the findings of this study are available from the corresponding author upon reasonable request.

## References

[1] Celakovsky P, Kalfert D, Smatanova K, Kordac P, Laco J, Chrobok V (2017). Discordance between clinical and pathological TNM classification: influence on results of treatment and prognosis in patients with laryngeal cancer. Neoplasma.

[2] Agada FO, Nix PA, Salvage D, Stafford ND (2004). Computerised tomography vs. pathological staging of laryngeal cancer: a 6-year completed audit cycle. Int J Clin Pract.

[3] Chang BA, Lott DG, Nagel TH, Howard BE, Hayden RE, Hinni ML (2019). Outcomes following transoral laser microsurgery with resection of cartilage for laryngeal cancer. Ann Otol Rhinol Laryngol.

[4] Forastiere AA, Ismaila N, Lewin JS, Nathan CA, Adelstein DJ, Eisbruch A, Fass G, Fisher SG, Laurie SA, Le Q-T (2018). Use of larynx-preservation strategies in the treatment of laryngeal cancer: American Society of Clinical Oncology clinical practice guideline update. J Clin Oncol.

[5] Kuno H, Sakamaki K, Fujii S, Sekiya K, Otani K, Hayashi R, Yamanaka T, Sakai O, Kusumoto M (2018). Comparison of MR imaging and dual-energy CT for the evaluation of cartilage invasion by laryngeal and hypopharyngeal squamous cell carcinoma. Am J Neuroradiol.

[6] Lefebvre J-L, Ang KK (2009). Larynx preservation clinical trial design: key issues and recommendations—a consensus panel summary. Int J Radiat Oncol Biol Phys.

[7] Harréus U (2013). Surgical errors and risks—the head and neck cancer patient. GMS Curr Top Otorhinolaryngol Head Neck Surg.

[8] Foote R, Gilbert J, Gillison M (2019) Continue NCCN guidelines panel disclosures. Version 2.2018 head and neck cancers. National Comprehensive Cancer Network website

[9] Phelps P (1992). Carcinoma of the larynx—the role of imaging in staging and pre-treatment assessments. Clin Radiol.

[10] Zbären P, Becker M, Läng H (1996). Pretherapeutic staging of laryngeal carcinoma: clinical findings, computed tomography, and magnetic resonance imaging compared with histopathology. Cancer.

[11] Zbären P, Becker M, Läng H (1997). Staging of laryngeal cancer: endoscopy, computed tomography and magnetic resonance versus histopathology. Eur Arch Otorhinolaryngol.

[12] Pietragalla M, Nardi C, Bonasera L, Mungai F, Verrone GB, Calistri L, Taverna C, Novelli L, Locatello LG, Mannelli G (2020). Current role of computed tomography imaging in the evaluation of cartilage invasion by laryngeal carcinoma. Radiol Med (Torino).

[13] Joshi VM, Wadhwa V, Mukherji S (2012). Imaging in laryngeal cancers. Indian J Radiol Imaging.

[14] Zan E, Yousem DM, Aygun N (2011). Asymmetric mineralization of the arytenoid cartilages in patients without laryngeal cancer. Am J Neuroradiol.

[15] Li H, Chen X (2017). Diagnostic value of enhanced CT/MRI for thyroid cartilage invasion by malignant tumor. Chin J Otorhinolaryngol Head Neck Surg.

[16] Foucher M, Barnoud R, Buiret G, Pignat J-C, Poupart M (2012). Pre-and posttherapeutic staging of laryngeal carcinoma involving anterior commissure: review of 127 cases. Int Sch Res Not.

[17] Koch WM, Ridge JA, Forastiere A, Manola J (2009). Comparison of clinical and pathological staging in head and neck squamous cell carcinoma: results from Intergroup Study ECOG 4393/RTOG 9614. Arch Otolaryngol Head Neck Surg.

[18] Freihat O, Pinter T, Kedves A, Sipos D, Cselik Z, Repa I, Kovács Á (2020). Diffusion-weighted imaging (DWI) derived from PET/MRI for lymph node assessment in patients with head and neck squamous cell carcinoma (HNSCC). Cancer Imaging.

[19] Roushdy MMM, Elsherif MMR, Kayed EMS, Farghaly S, Sayed ARJ (2022). Does diffusion magnetic resonance imaging (DWI) has role in irradiated laryngeal carcinoma?. Indian J Otolaryngol Head Neck Surg.

